# Do intuitive ideas of the qualities that should characterize involuntary and voluntary memories affect their classification?

**DOI:** 10.1007/s00426-020-01465-3

**Published:** 2021-02-13

**Authors:** Krystian Barzykowski, Giuliana Mazzoni

**Affiliations:** 1 Applied Memory Research Laboratory, Institute of Psychology, Jagiellonian University, ul. Ingardena 6, 30-060 Kraków, Poland; 2 Università di Roma, La Sapienza, Italy

## Abstract

It is assumed that the difference between voluntary and involuntary autobiographical memories lies in the intentionality to retrieve a memory assigned by the experimenter. Memories that are retrieved when people are instructed to do so in response to cues are considered voluntary (VAMs), those that pop up spontaneously are considered involuntary (IAMs). VAMs and IAMs so classified are also found to differ in terms of phenomenological characteristics, such as perceived accessibility, vividness etc. These differences are assumed to be due to differences in intentionality and the different retrieval processes at play. It is possible, however, that these differences (which are subjective attributions of phenomenological characteristics) are the result of metacognitive beliefs of what IAMs and VAMs should be. In two experiments, we investigated the possible role of these metacognitive beliefs. Participants rated IAMs and VAMs on a number of phenomenological characteristics in two conditions, when these memories were presented in blocks that specified whether they were retrieved in a voluntary or involuntary task, or when presented in a mixed list with no information provided. If metacognitive beliefs influence the reporting of memory properties, then the block presentation would increase the differences between the characteristics of the two types of memories. The results showed that, besides replicating the characteristics of IAMs and VAMs already observed in the literature, there were almost no differences between the blocked and the mixed lists. We discuss the results as supporting the idea that the difference in characteristics attributed to IAMs and VAMs reflect a genuine difference in the nature of the retrieval and is not the result of pre-existing metacognitive belief on what a voluntary and an involuntary memory should be.

## Introduction

When studying autobiographical memory (people’s memories of their personal past, see Conway & Pleydell-Pearce, 2000; Berntsen & Rubin, 2012), two types of retrieval are considered, voluntary and involuntary. While the former is the result of an intention to retrieve a given memory and typically (but not always, e.g. Barzykowski & Staugaard, 2016, 2018; Barzykowski, Niedźwieńska, & Mazzoni, 2019a, b, c; Harris et al. 2015; Uzer, Lee, & Brown, 2012) involves an effortful search (Botzung, Denkova, Ciuciu, Scheiber, & Manning, 2008; Conway & Loveday, 2010; Hall, Gjedde, & Kupers, 2008; Haque & Conway, 2001), involuntary memories (henceforth throughout the paper called also IAMs) come to mind without any conscious and explicit attempt to retrieve (Berntsen, 1996, 2010). As a result, involuntary memories are perceived as being retrieved with minimal (if any) cognitive effort and as unexpected, while voluntary memories (henceforth throughout the paper called also VAMs) are expected and the result of varying degrees of effort. Each time we try to recall something from our past (e.g. *whether we have extended our monthly pass for the public transport*), we retrieve voluntary autobiographical memories, while involuntary memories pop into our mind without any preceding intention to retrieve (e.g. while washing the dishes *the moment when we were extending our monthly pass* pops in our mind unexpectedly).

Over the years, there have been several shifts in the literature on the nature of autobiographical memories. As a result, involuntary memories are now treated as (1) a phenomenon worthy of investigating in and outside the laboratory (e.g. Berntsen, 1996; Roberts, McGinnis, & Bladt, 1994; Schlagman and Kvavilashvili, 2008) and (2) a basic mode of remembering (e.g. Berntsen, 2010, 2015; Brewin, Gregory, Lipton, & Burgess, 2010; Clark, Mackay, & Holmes, 2013; Moulds & Krans, 2015).

Although for several years there was a strong distinction in the memory literature between involuntary and voluntary memories in terms of both retrieval intentionality and/or retrieval effort, recent studies have challenged this position (e.g. Barzykowski & Staugaard, 2016, 2018; Barzykowski et al., 2019a, b, c; Jeunehomme & D'Argembeau, 2015; Uzer & Brown, 2017; Uzer et al., 2012) by showing that—similarly to involuntary memories—people frequently retrieve voluntary memories also with little cognitive effort. Thus, in a narrow sense, it may be argued that retrieval effort does not entirely differentiate between involuntary and voluntary memories (Barzykowski & Staugaard, 2016; see however Barzykowski, Staugaard, & Mazzoni, 2021; Sanson et al., 2020). Retrieval intentionality (typically decided by the experimental instructions) seems then to remain the main factor responsible for the distinction.

A common result is that VAMs and IAMs differ on a number of phenomenological characteristics rated by the participants. For example, IAMs are typically found to be rated as more accessible, more specific, more vivid, as well as more clear, emotionally intense, personal, important (just to name a few of the characteristics) compared to VAMs (e.g. Barzykowski & Staugaard, 2016, 2018; Barzykowski et al., 2019a, b, c; Schlagman & Kvavilashvili, 2008; Staugaard & Berntsen, 2014). This distinction has been taken as confirmation of the different nature of the retrieval processes responsible for the two types of memories. Although additional more objective confirmation of the different processes involved in IAMs and VAMs comes from recent studies showing that they also differ in terms of the retrieval latencies (e.g., Barzykowski & Staugaard, 2016, 2018; Barzykowski et al., 2019a, b, c; Cole, Staugaard, & Berntsen, 2016; Johannessen & Berntsen, 2010; Schlagman & Kvavilashvili, 2008), it is not clear how the phenomenological differences can be explained. Berntsen (2009) suggested that involuntary retrieval favours memories that are highly accessible (e.g., novel and emotional). Building on this idea, it has been recently proposed that also for IAMs, each memory has to pass an awareness threshold^1^ in order to reach one’s consciousness (threshold hypothesis, Barzykowski & Staugaard, 2016, 2018; Barzykowski et al., 2019a, b, c). According to this hypothesis, ease of retrieval is possibly linked to the perceived phenomenological characteristics of the memory. For instance, it may be easier for a phenomenologically ‘*juicy*’ memory (e.g. that is highly vivid, emotionally intense) to pass the awareness threshold because such memory property may be especially good at drawing one’s memory-related attention (see also Barzykowski & Staugaard, 2016, 2018; Barzykowski et al., 2019a, b, c).^2^ As priming effects show, because memories differ in their accessibility, it is more likely that highly rather than weakly activated memories will enter awareness (for an example of studies on priming voluntary and involuntary memories, see Ball & Hennessey, 2009; Barzykowski & Niedźwieńska, 2018a; Mace, 2005; Mace & Clevinger, 2013; Mace & Unlu, 2020).

The accessibility of memories can be modified by various factors such as emotional intensity, retrieval effort, importance, vividness, rehearsal, recency, and usualness (Barzykowski & Staugaard, 2018; Ritchie, Skowronski, Walker, & Wood, 2006). We conceive awareness threshold as the minimum amount of activation of a memory that helps the memory to become aware. Entering awareness can be achieved either when a memory reaches levels of activation that are greater than a given threshold, or when the threshold is lowered by factors such as expectations, etc. As pointed out by Reed (2007, p. 49) a key feature of the threshold is that it may be momentarily modified by different factors (e.g. expecting something to happen, placing the focus of attention on only some type of stimuli), which may increase the likelihood of specific stimuli entering awareness. Building on this idea, the threshold hypothesis states that, while both highly and poorly accessible memories can be retrieved either voluntarily or involuntarily, the processes operating during memory retrieval can influence the frequency of each type of retrieval by increasing or lowering the awareness threshold. Barzykowski and Staugaard (2018; also, Barzykowski et al., 2019a, b, c) proposed these processes to be retrieval intention (i.e. wanting to retrieve a memory) and selective monitoring (i.e. expecting a memory to appear). In the threshold hypothesis, a memory’s accessibility is therefore not determined only by retrieval or encoding processes, but by a complex interplay between factors during encoding (i.e. how intense or important the episode was), during consolidation (e.g. how efficiently the memory was integrated within the memory system), and during retrieval (e.g. if the focus of attention is placed on the retrieval of a given memory). Intention and monitoring can be conceived as processes that enable access to otherwise less accessible memories.^3^ As a result, compared to involuntary memories, voluntary memories show several indicators of lower accessibility (i.e. low emotional intensity and low personal significance).

Yet, another plausible group of factors that should be considered and, importantly, that may also determine the phenomenological characteristics rated by participants are people’s metacognitive beliefs and lay preconceptions about how different types of memories should be. Therefore, it may be that involuntary memories are rated as clear, very emotionally intense and insightful not only because of their intrinsic, objective properties but also because of the naïve belief associated to the experience (e.g., ‘*memories retrieved without intention are special and different’*). Since there are no studies showing the possible influence of metacognitive beliefs on the rating of phenomenological memory characteristics, one cannot rule out such a possibility. We discuss this in more details below.

### The possible effects of metacognitive beliefs on autobiographical memory retrieval

When considering memory properties rated by participants and use them as an indicator of the difference between involuntary and voluntary memories, two issues need to be considered. The first is whether all memories reported by participants as ‘involuntary’ are indeed retrieved without the participant’s intention. The second is to assess whether the ratings of phenomenological characteristics are based on the objective properties of the memories, or on what participants believe the characteristics of involuntary memories should be.

The first is a methodological issue. In experimental psychology, autobiographical memory has most often been investigated using the word-cue method (Crovitz & Schiffman, 1974), where participants are presented with a number of verbal cues and asked to deliberately recall a personal episode in response to a cue (e.g. Barzykowski et al., 2019a, b, c). This word-cue method was also adapted by Schlagman and Kvavilashvili (2008) for the first experimental procedure of studying involuntary memories. In it, participants were asked to perform a boring attentional task while watching short cue phrases on a computer screen, some of which may incidentally trigger involuntary memories. Importantly, participants were specifically instructed to report when unexpectedly autobiographical memories were coming to mind during the task. While this allowed for the recording of involuntary memories under well-controlled experimental conditions, informing participants that they had to report only involuntary memories might have on one hand triggered monitoring processes that might have interfered with the unintentional retrieval, on the other hand the retrieval might have been at least occasionally intentional. The risk then is that this experimental procedure alters the involuntariness of the retrieval by either priming autobiographical memories and/or inducing voluntary processes (for more one the possible effects of different type of instructions see Barzykowski & Niedźwieńska, 2016; Vannucci et al., 2014). Thus, the core of this methodological challenge is to instruct participants to report involuntary memories without changing how these memories are *naturally* retrieved (see also Barzykowski, 2014; Barzykowski & Niedźwieńska, 2012, for a similar argument). This is especially important given the fact, as already argued by Michael, Garry, and Kirsch (2012), that expectations of a particular outcome, (e.g. the expectation of experiencing involuntary memories) may automatically modify our cognitive processes and behaviour to produce that outcome. So far several solutions have been devised in the attempt to minimize the unwanted influence of this ‘observer effect’ on the involuntariness of retrieval. Among them, for example, (a) the usage of probe-caught methods (e.g. Barzykowski et al., 2019a, b, c; Batool & Mazzoni, 2011; Mazzoni, 2019; Vannucci et al, 2014, 2019; Plimpton et al. 2015), (b) the usage of effort control scales (e.g. Barzykowski & Niedźwieńska, 2016; Barzykowski & Staugaard, 2016, 2018), (c) instructing participants to report any mental content without placing the focus of attention only on retrieval of autobiographical memories (Batool & Mazzoni, 2011; Vannucci et al, 2014), (d) when asking about the phenomenological properties of memories, keeping the rating procedure brief in order to not to interfere with the natural flow of involuntary memory retrieval applied by the usage of the two-step rating procedure described below (Batool & Mazzoni, 2011).

The second issue relates to relying on the participants’ introspection, in general, and on participants’ subjectivity, in particular, in deciding which memory is involuntary or voluntary, and in rating the memory phenomenological characteristics (e.g. vividness, clarity, emotional intensity). Conclusions about autobiographical memory retrieval, in general, and involuntary vs voluntary memories, in particular, which are based on phenomenological characteristics may be especially influenced by participants preconceptions and metacognitive beliefs about remembering their personal past. Given that we do not have any objective indicators of these memory properties yet, we typically “trust” participants’ judgments and their responses. However, there is the need to assess the extent to which these ratings are influenced by the belief participants hold about the nature and characteristics of involuntary memories.

### Metacognitive belief

Beliefs about memory are also referred to as *laypeople theories*, *naïve theories*, *implicit theories*, *folk theories*, or *mindsets*; e.g. Zedelius & Schooler, 2017). They are ultimately metacognitive beliefs, where metacognition has been defined as “*cognition about cognition*” (Flavell & Ross, 1981) or “thinking about thinking” (Yussen, 1985). More specifically, in this context it refers to the “stable knowledge or beliefs about one ‘s own cognitive system, and knowledge about factors that affect the functioning of the system; the regulation and awareness of the current state of cognition, and appraisal of the significance of thought and memories” (Wells, 1995, p. 302). As a result, metacognitive beliefs help people to interpret and understand both their own and other people’s cognitive behaviour (Dweck, Chiu, & Hong, 1995). While in general they may be useful, they may be sometimes also irrational and unreasonable (Palmier-Claus, Dunn, & Lewis, 2011). Metacognitive beliefs may have significant effects on a wide range of phenomena, including emotion maintenance and regulation (e.g. Tajrishi, Mohammadkhani, & Jadidi, 2011), memory performance (e.g. Horhota et al., 2012; Irak & Çapan, 2018), social functioning (Bright et al. 2018), spontaneous thinking (for a review see Morewedge & Kupor, 2018), and well-being (Sellers, Varese, Wells, Morrison, 2017; Østefjells et al., 2017), to name just a few.

Interestingly, the role of people’s lay theories was already extensively studied in mind wandering (for a review see Zedelius & Schooler, 2017), and involuntary memories may constitute the content of at least some of the task-unrelated thoughts studied in mind wandering research (for similar views see Johannessen & Berntsen, 2010; Plimpton et al., 2015; Mazzoni, 2019). For instance, the more control people believe they have over their mind wandering, the less they mind wander, and importantly, this is also true for experimentally induced/manipulated belief (Zedelius & Schooler, 2017; Zedelius, Protzko, & Schooler, 2017, 2020). In addition, in a series of experiments on spontaneous thoughts Morewedge, Giblin, and Norton (2014) provided strong evidence that the perceived lack of control over spontaneous thoughts (which, as a reminder, resides at the heart of the involuntary and voluntary memory distinction) leads people to perceive them as more meaningfully self-insightful. As a consequence, when thoughts appeared to have been retrieved involuntarily rather than voluntarily, people believed them to provide more meaningful and important insight about their own self and had potentially higher impact on their judgements. This series of results would strongly suggest that metacognitive beliefs influence autobiographical memory retrieval, and in particular the rating of involuntary memories characteristics. Involuntary memories might be even more subjected to the influence of beliefs as there is no control over and access to the involuntary memory retrieval.^4^

Strikingly, whereas over the last several years much progress has been made in gaining a better understanding of involuntary and voluntary autobiographical memory retrieval, the role of metacognitive beliefs in creating the distinction between involuntary and voluntary memory retrieval has scarcely been investigated. To the best of our knowledge there is so far only one published study (i.e. Sanson et al., 2020) addressing the possibility that “voluntary” versus “involuntary” retrieval may be indeed an attribution-based process suggesting that it may be also influenced by metacognitive beliefs.^5^

### The present study

In summary, previous research show that people may ascribe different attributes to the memory retrieval which suggest that some part of the memory retrieval may be an attributional-like process. In addition, there is now also a large body of evidence showing that metacognitive beliefs may influence many aspects of our every-day functioning. However, no prior study investigated their role in the subjective distinction between involuntary and voluntary autobiographical memory retrieval.

The main goal of the present studies was to address the question concerning the possible influence of metacognitive beliefs on phenomenological memory properties. We manipulated retrieval intentionality (voluntary = recall a memory for each cue; involuntary = report any involuntary memory that comes to mind spontaneously). We investigated voluntary autobiographical memories using the word-cue method (Crovitz & Schiffman, 1974), in which an individual is presented with verbal cues and asked to recall a personal memory in response to each cue. To study involuntary memories, we used a modified version of Schlagman and Kvavilashvili’s (2008) experimental design, which allowed us to control the retrieval phase and observe memories retrieved without explicit intention. This an often-employed experimental methodology devised by Schlagman and Kvavilashvili (2008) to elicit involuntary memories under well-controlled experimental conditions (see for example Barzykowski & Staugaard, 2016, 2018; Barzykowski & Niedźwieńska, 2016, 2018a, b; Barzykowski et al., 2019a, b, c; Mazzoni, Vannucci, & Batool, 2014; Vannucci et al., 2014). More precisely, this task employed a two-step procedure of rating the memory content. First, while performing the vigilance task (detecting infrequent target vertical lines in a stream of slides with horizontal lines), participants were instructed to write down any spontaneously occurring memories experienced. In addition, they also rated the retrieved memories on few phenomenological characteristics (Part 1: e.g. vividness, clarity, intensity of emotions). This ‘online’ rating procedure was deliberately brief in order not to interfere with the main vigilance task. Second, after completing the main task, participants were presented with their previously recorded memories and asked to rate the characteristics of the event that each memory referred to using a larger number of questions (Part 2: e.g. importance of the event, personal nature, rehearsal: recalling in the past). Importantly, in the present study we separated these two phases using a filler task between the ‘online’ and post-task rating procedures (see below for more details). Thanks to this two-step procedure it is possible to distinguish between ratings based on characteristics perceived during retrieval (Part 1: The online-rating procedure) and characteristics assigned afterwards, that we hypothesized are based on metacognitive beliefs (Part 2: The post-task rating procedure).

To assess the role of metacognitive belief, besides expecting a greater effect of belief in the post-task rating procedure, we also manipulated the presence/absence of information about the retrieval intentionality at the time of the phenomenological characteristic rating of the memories. For the rating, participants were provided with either (1) separated lists of the memories they had reported, clearly and correctly labelled as involuntary or voluntary; or (2) one mixed list containing in random order both involuntary and voluntary memories, which were not labelled. Ratings were requested after a delay in order to minimize the possibility that participants remembered the intentionality of memory retrieval. Participants who received the labelled separate lists were in the “specified memory origin” group. The participants who received the mixed list were in the “unspecified memory origin” group. In general, we argue that any metacognitive beliefs about involuntary and voluntary memories should be especially strongly activated when people are informed of whether the memory was retrieved voluntarily or involuntarily (‘specified memory origin’ group) and minimized when people are not informed of the voluntary or involuntary nature of retrieval (‘unspecified memory origin’ group). Therefore, if beliefs about the nature of retrieval play a role, then we should observe stronger differences in phenomenological characteristics between involuntary and voluntary memories in the ‘specified memory origin’ group compared to the ‘unspecified memory origin’ group. The straightforward idea along these lines is that since in previous studies involuntary and voluntary memories were always clearly labelled and significant differences were found in phenomenological characteristics, we do not know whether these observed differences were due to actual differences in memory characteristics, or to the belief about which qualities should characterize involuntary and voluntary memories. If this is true that beliefs play a major role, then involuntary and voluntary memories should not be rated so differently when their origin is not highlighted at all. Our study is thus the first to examine the phenomenological characteristics of involuntary and voluntary memories when minimizing the information about the origin (voluntarily or voluntarily retrieved) of a memory, and thus to minimizes the potential role of metacognitive belief.

Overall, we expected that memories retrieved involuntarily (i.e. during the involuntary retrieval phase) would be in general more accessible compared to voluntary memories, and therefore more quickly retrieved. Phenomenologically, we expected to replicate the results as observed in previous studies (e.g. Barzykowski & Staugaard, 2016, 2018; Barzykowski et al., 2019a, b, c), showing that involuntary memories would be perceived more effortless, vivid and clear, accompanied by physiological sensation, emotional intensity, relevance to a person’s current life situation. We also expected, and this is the main hypothesis, the difference between phenomenological characteristics of involuntary and voluntary memories to be significantly lower in the unspecified memory group than in the specified memory group. This result was expected in particular in the post-task rating.

## Study 1

The Research Ethics Committee at Jagellonian University approved both Study 1 and Study 2 (no. KE/01/102,018). Written consent for participation was obtained prior to data collection.

### Design

We employed a mixed design. Task (involuntary memory retrieval vs. voluntary memory retrieval) was the within-subjects factor. We called memories reported during the involuntary task ‘involuntary memories’ and memories reported during in the voluntary task ‘voluntary memories’. In other words, we assumed the two tasks to differ substantially in retrieval intentionality and operationalized the retrieval intentionality as the conscious decision to retrieve a memory. The other factor in the design (between-subjects) was information about the origin of the memories for the phenomenological characteristics rating (specified memory origin group vs. unspecified memory origin group). While participants in the specified memory origin condition were explicitly informed about the type of memories to be rated and told whether the memory was from the voluntary or involuntary task, participants in the unspecified memory origin group rated memories presented in a random order in a mixed list, without being told whether voluntary or involuntary. We expected participants in this group did not pay explicit attention to the nature (voluntary or involuntary) of the memories. We assessed the influence of this information on the phenomenological characteristics reported by participants.

### Participants

A total of 60 participants (44 females, *M*_age_ = 23.42, SD = 4.28, range 19–39 years; one participant did not indicate age) were recruited and randomly assigned to the two experimental groups (specified memory origin and unspecified memory origin). Participants were tested in groups of two to twelve in a laboratory with separate computer stations. Five participants did not report any involuntary autobiographical memory, and additional seven had less than 50% of correct responses on the vertical lines task, and their results were excluded from the analysis. The final sample consisted of 48 participants with 24 participants in the specified memory origin group (18 females, *M*_age_ = 23.66, SD = 4.99, range 20–39 years), and 24 participants in the unspecified memory origin group (19 females, *M*_age_ = 23.19, SD = 3.08, range 19–32 years). They participated in return for a 20 PLN (ca. 5 USD).

### Materials

#### The Involuntary Memory Programme (IMP)

We employed the Involuntary Memory Programme (IMP) (for a complete description of the programme, see also Barzykowski & Niedźwieńska, 2016, pp. 5–6; Barzykowski & Staugaard, 2016, p. 524) that was successfully used in previous studies on involuntary and voluntary memory retrieval (e.g. Barzykowski & Niedźwieńska, 2018a, b; Barzykowski et al., 2019a, b, c). This is a modified and fully computerized task based on Schlagman and Kvavilashvili’s method (2008; used also by, Mazzoni et al., 2014; Vannucci et al., 2014). The main differences between the current task and Schlagman and Kvavilashvili’s (2008) original design were as follows: (1) using 400 slides instead of 800, (2) use of a computerized version of all scales and questions, (3) extending the presentation of each trial from 1.5 to 2 s, and (4) (only in Study 2 of the present paper) instructing participants to write down any involuntarily occurring thoughts instead of reporting only autobiographical memories.

The vigilance task involved detecting patterns of vertical lines (seven target slides) in a stream of 393 non-target slides with horizontal lines. Slides were presented for 2 s with short verbal phrases (e.g., *driving a bike*, *romantic dinner*) displayed in the centre of each slide. They acted as potential triggers for involuntary memories (Study 1) or involuntary thoughts (Study 2). There was an approximately equal number of neutral (*N* = 134; e.g. *buying a bread, putting on pants*), positive (*N* = 133; e.g. *receiving a present, a wonderful smile*), and negative (*N* = 133; e.g. *unpleasant conversation, lost wallet*) phrases, that constituted the final pool of 400 phrases, which were randomly selected from the pool of 800 phrases used in previous studies (e.g., Barzykowski & Niedźwieńska, 2016, 2018a; Barzykowski & Staugaard, 2016, 2018; for details about the Polish adaptation see also Barzykowski & Niedźwieńska, 2016, p. 6). From the rest of 400 cues (i.e. that were not selected to be used in the involuntary memory recording phase) we randomly selected new 16 word phrases (5 positive, 5 negative and 6 neutral) that were used as cues in the voluntary memory recording phase.

*Equivalence of cues between phases* To investigate the comparability of cues used in the involuntary and voluntary memory recording phases, all cues were rated for imagery, concreteness, and typicality on 7-point scales (1 = *low* to 7 = *high*) by independent 10 participants. The mean ratings for cues used in the involuntary and voluntary phases were entered into three separate *t*-tests for independent samples with concreteness, imagery, and typicality as dependent variables. There were no significant main effects (*p* > 0.13) of cue type (cues used in the voluntary vs involuntary phases) for any of the characteristics. Therefore, we argue that any differences between reported memories in the present study are not due to differences in the characteristics of the verbal phrases used in the voluntary and involuntary phases.

#### The positive and negative affect schedule (PANAS; Brzozowski, 2010)

This scale measures the strength of negative and positive emotions and consists of 30 items measuring current emotional states. Participants have to rate on a five-point scale the extent to which the given adjectives correspond with their current state. The reliability coefficients (internal consistency and stability) of the Polish version of the PANAS range from 0.73 to 0.95 (Brzozowski, 2010). It was used to control for possible differences between the conditions. The PANAS was used twice during the study to test and to control the comparability of groups and study phases. The first one, was at the beginning of the study. The second time was just after the filler task just before the post-task rating procedure.

#### The social desirability scale (Drwal and Wilczyńska, 1980)

The Social Desirability Scale (Drwal and Wilczyńska, 1980) is a self-report tool for measuring an individual’s need to be accepted and being ready to behave in a manner that is perceived favourably by others. The scale consists of 29 items of the “true–false” type (e.g. *I am never late for school/work*). The reliability coefficients (internal consistency and stability) of the questionnaire equalled 0.79–0.90. High coefficients of correlation (up to 0.82) with Marlowe-Crowne’s scale (Crowne and Marlowe, 1960) were also obtained (Drwal and Wilczyńska, 1980). This way, as it was also done by Schlagman and Kvavilashvili (2008), we wanted to find an indirect indicator of assess the possibility that participants tried to deliberately recall mental contents to please the experimenter.

#### The squire subjective memory questionnaire (SSMQ; Kuczek, Szpitalak, & Polczyk, 2018)

The Squire Subjective Memory Questionnaire (SSMQ; Squire, Wetzel, & Slater, 1979) is a self-report tool for measuring an individual’s trait memory distrust. The scale consists of 18 statements (e.g. *My ability to remember what I read and what I watch on television is…*) rated on a 9-point scale ranging from − 4 (*disastrous*) to + 4 (*perfect*). The reliability coefficients (internal consistency and stability) of the questionnaire were 0.87–0.89. Significant coefficients of correlation (up to 0.57) with main scales of the Memory Assessment Clinics Self-Rating Scale—Revised (Crook & Larrabee, 1990; Winterling, Crook, Salama, & Gobert, 1986; Polish adaptation: Doromoniec, 2004) were also obtained. The higher SSMQ scores the higher trust in a memory one has. It was used to control for possible differences between groups in subjective memory evaluation.

#### Filler tasks

After finishing the voluntary memory recording phase and before starting the post-task rating procedure participants in both conditions played for 10 min a few games selected from CONCENTRATION Part 2. Mind Academy software (similarly to previous studies, e.g. Barzykowski & Niedźwieńska, 2018a). The break was used to divide the post-task rating procedure from previous involuntary and voluntary memories recording phases. These exercises engage: ability to analyse stimuli and information, constructive problems solving, inductive and deductive reasoning. They were set on a low level of difficulty without time pressure. The material in the games was rather abstract and non-verbal. Therefore, it is rather unlikely that it might have involuntarily or voluntarily triggered any episodic or autobiographical memories from the personal past.

#### Procedure

Participants were tested in groups of two to twelve and were free to withdraw from the study at any time. Each experimental session consisted of four phases. The first one was a vigilance task during which participants recorded involuntary memories. The second one, following immediately after the first one, was a voluntary memory phase. We specifically did not counterbalance the order of the phases and we started with the involuntary phase first in order to keep our participants unaware of the true goal of our study (autobiographical memory retrieval) and to decrease the amount of voluntary memories during the latter phase due to a carry-over effect if we started with voluntary phase first. In the third phase, participants were engaged in the filler tasks for 10 min. Finally, the fourth phase was the post-task rating procedure during which participants were provided with memories reported during the first and second phase, either separately in involuntary and voluntary blocks (the specified memory origin group) or randomly within one block (the unspecified memory origin group). They also described the memories more thoroughly and rated them on an additional number of phenomenological characteristics. These phases are presented more thoroughly below.

*Involuntary memory recording phase* Just before starting the IMP, participants filled in the Positive and Negative Affect Schedule (PANAS; Brzozowski, 2010). Next, as in previous studies (e.g. Barzykowski & Staugaard, 2018, restricted conditions; Barzykowski & Niedźwieńska, 2016; Barzykowski et al., 2019a, b, c, restricted conditions; Schlagman & Kvavilashvili, 2008; Vannucci et al., 2014; Mazzoni et al., 2014; Mazzoni, 2019), participants were informed that since the vigilance task might be dull, they might experience different kinds of thoughts to pop in their mind during the task. We provided them with examples of such thoughts, including personal goals, words, current concerns, plans, and memories. Importantly, in this study participants were instructed to report only autobiographical memories that spontaneously came to mind during the vigilance task. In addition, we emphasized that memories can be general or specific. All participants were instructed to write down any involuntary memory that occurred during the 400 vigilance trials by pressing the spacebar as soon as they became aware of one coming to mind and typed them into the computer programme, regardless of what it was or how interesting they found it to be. They could refrain from reporting sensitive memories by typing “X” as an answer, or by providing a general description. After pressing the spacebar, they briefly described the memory and rated it on a 7-point scale on the following dimensions: (1) the extent to which the content was accompanied by unexpected physiological sensations (henceforth, called physiological sensation), (2) the extent to which they had deliberately tried to bring the thought to mind (henceforth, called effort), (3) the intensity of emotions experienced in response to the content, (4) the vividness of the memory (i.e. feeling of reliving), (5) clarity (i.e. how clearly and well an individual remembered a given memory), (6) how specific and concrete the content was, and (7) how personal the memory was. All points along the scales were clearly labelled during the task. As an example, the scale for the effort was as follows: (1 = *I wasn’t trying at all*, 2 = *I wasn’t trying*, 3 = *I don’t think that I tried*, 4 = *I tried a little bit*, 5 = *I tried somewhat*, 6 = *I tried*, 7 = *I tried very hard*). Participants also mentioned if the memory occurred deliberately (they decided to think about it) or involuntarily (it simply popped in their mind),^6^ what triggered the memory (1 = *Something in the programme*, 2 = *Something in my mind*, 3 = *Something in the surroundings*, 4 = *Nothing*) and provided a brief description of the trigger. Henceforth, this part of the procedure will be referred to as the *online rating*. After answering these questions for each memory reported, participants clicked ‘continue’ to return to the vigilance task. After the completion of the vigilance task, the programme stopped, and the experimenter briefly introduced the participants to the second phase of the procedure by providing verbal instructions about how to complete the voluntary memory phase.

*Voluntary memory recording phase* In the voluntary memory recoding phase, participants were provided with oral and written instruction concerning the nature of autobiographical memories. It was explained that memories could be specific or general and recent or remote. They were instructed to recall a past memory as quickly as possible in response to the verbal phrase displayed on the screen, without omissions. As soon as they retrieved a memory, they should press the spacebar. If participants did not press the spacebar within 60 s, the programme automatically proceeded to the next phrase. After pressing the spacebar, they would then immediately provide a brief description of the memory and answer the same questions as for the online rating of involuntary memories. After responding to the 16 cues, the programme was automatically stopped, and the filler tasks were administered. After completing the filler tasks and just before the fourth phase, participants filled in PANAS again (Brzozowski, 2010). Then, the experimenter provided participants with verbal and written instructions describing the fourth phase; namely, the post-task rating procedure.

*Post-task rating (reminding of the memory origin).* In the post-task rating participants were asked to answer additional questions relating to all the memories they reported during the previous two phases, starting with memories reported only during the vigilance task. Instructions were different for the specified and unspecified memory origin groups. In the former participants were reminded that the memories in the involuntary block were retrieved during the vigilance task spontaneously, effortlessly and without any retrieval intention, hence they were called ‘involuntary memories’. It was explicitly stressed that they would be next provided only with such ‘involuntary memories’. This way, we wanted to make participants pay explicit attention to the origin of the memory and activate and facilitate any existing metacognitive beliefs about voluntary memories and their characteristics. Then, memories were displayed one after the other in the same order as they had been recorded. Participants were instructed to read each memory and to click the ‘start’ button to initiate answering a series of questions. Participants rated on 7-point scales: (1) the extent to which they had deliberately tried to bring the memory to mind during the recording phase (same question as for online-rating procedure), (2) how detailed the memory was, (3) the vividness of the memory (same question as for the online-rating procedure), (4) the clarity of the memory (same question as for the online-rating procedure), (5) how personal the memory was (same question as for the online-rating procedure), (6) the perceived importance of the original event, (7) the emotional valence (i.e. how pleasant the event was), (8) the relevance of the memory to the participant’s identity, (9) the intensity of emotions that accompanied the original event, (10) how unusual the remembered event was, and (11) how often the memory had been recalled in the past (i.e. rehearsal). Participants also indicated their age when the event occurred and specified whether the memory was general or specific by classifying the event as: (1) extended in time (e.g. *when I was a undergraduate student*); (2) repeated in the past (e.g. *regular lectures*); or (3) relating to a particular situation that happened on a particular day (e.g. *the day I ate squid for the first time*). Both 1 and 2 were then classified as general events, while 3 was classified as a specific event.

After rating all involuntary memories, participants were told that they will be provided with memories reported during the voluntary memory task. They were reminded that these memories were retrieved in response to verbal cues displayed on the screen and they were retrieved in a voluntary and intentional fashion. Hence the name ‘voluntary memories’. It was explicitly highlighted that they would be next provided only with such ‘voluntary memories’. This way, we wanted to activate and facilitate any existing metacognitive beliefs about voluntary memories and their characteristics and to make participants paying explicit attention to the origin of the memories. Then, the memories were displayed one after the other in the same order as they had been recorded and participants answered the same post-task rating questions as described for involuntary memories.

Once participants rated all memories the unexpected recognition task was launched. More precisely, participants were provided one after the other with all memories recorded across the two phases in a random order and they were asked to decide for each memory in which phase it was reported (involuntary vs voluntary) by selecting a response on the screen. This was done also to control for possible differences between conditions in terms of how well participants may remember involuntary and voluntary memories. At the completion of this task, participants filled in the SSMQ (Kuczek, Szpitalak, & Polczyk, 2018) and the Social Desirability Scale (Drwal and Wilczyńska, 1980).

For the unspecified memory origin group, the only difference was that for the post-task rating procedure the voluntary and involuntary memories reported during the first and second phase were presented in random order within one block with no information about their type of retrieval. More precisely, participants were told that they would be provided with memories reported during the previous two phases in a random order but without indicating which memory had been retrieved in which phase. Withholding the information minimized the possibility that participants would pay explicit attention to the nature (voluntary or involuntary) of the memories, thus the likelihood of using beliefs about characteristics should play a lesser role.

### Results

#### Equivalence of experimental conditions

To test the comparability of groups, the overall participants’ means for the Social Desirability Scale, the Squire Subjective Memory Questionnaire, the vigilance task performance were entered into independent *t*-test. As can be seen in Table 1, no differences were observed between the two groups on any of these variables (*p* > 0.169).

**Table 1:** Means and standard deviations for variables measuring social desirability, mood, subjective memory trust, vigilance task performance across groups in Study 1 and Study 1

				Study 1							Study 2					
				Group							Group					
	Specified Memory Origin		Unspecified Memory Origin						Specified Memory Origin		Unspecified Memory Origin					
	*M*	SD	*M*	SD		*t*	*p*	*d*	*M*	SD	*M*	SD		*t*	*p*	*d*
Social Desirable Scale	14.46	5.36	12.83	4.49		1.13	0.265	0.33	12.90	5.17	11.76	3.69		0.88	0.387	0.25
The Squire Subjective Memory Questionnaire	120.79	20.84	113.09	15.53		1.43	0.159	0.42	117.95	18.12	116.12	16.22		0.36	0.719	0.11
Vigilance Task Performance	0.85	0.13	0.81	0.16		0.98	0.333	0.27	0.86	0.15	0.80	0.17		1.18	0.246	0.37
PANAS: Positive affect_1	47.08	10.66	42.87	9.16		N/A			47.00	12.59	42.24	9.04		N/A		
PANAS: Positive affect_2	40.13	13.92	37.96	11.58					43.86	10.43	37.64	13.03				
PANAS: Negative affect_1	18.79	4.97	18.39	5.06					20.24	6.44	20.04	7.26				
PANAS: Negative affect_2	17.46	3.92	16.39	2.04					18.57	4.18	17.60	5.46				
The accuracy of involuntary memory recognition	0.69	0.33	0.72	0.22					0.79	0.20	0.71	0.29				
The accuracy of voluntary memory recognition	0.80	0.18	0.70	0.19					0.85	0.22	0.75	0.26				

Next, the overall means for the mood scores measured by PANAS were entered into two separate 2 condition (specified memory origin, unspecified memory origin) × 2 time of testing (before the vigilance task, before the post-task rating procedure) mixed ANOVA with repeated measures on the last factor. The analysis revealed a significant main effect of time of testing on the positive, *F*(1, 45) = 20.73, *p* < 0.001, $\eta_{{\text{p}}}^{2}$ = 0.32, and negative affect, *F*(1, 45) = 7.69, *p* < 0.008, $\eta_{{\text{p}}}^{2}$ = 0.15. More precisely, before the post-task rating procedure participants had lower ratings on both positive and negative affect scales compared to the beginning of the experimental session (see Table 1). It is highly possible that performing a monotonous vigilance task and voluntary conditions somehow changed the mood of participants (i.e. they felt less nervous and stressed as well as less active and lively). However, neither the main effect of group, nor the group by phase interaction were significant (*p* > 0.307; *F* < 1.07).

**Fig. 1:**
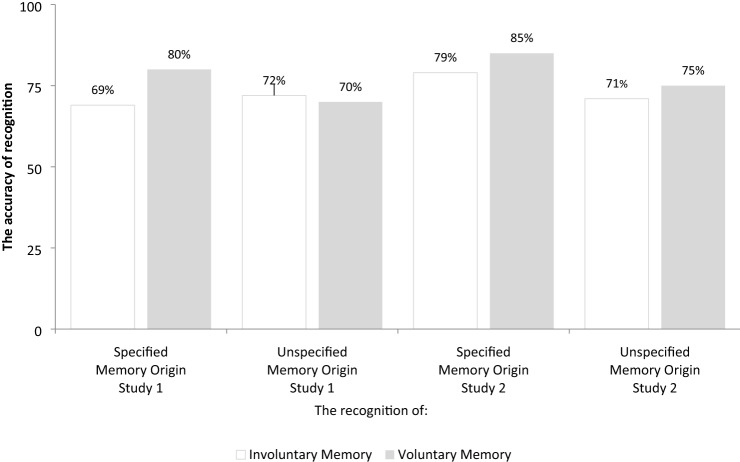
The percentage of accuracy of recognition of involuntary and voluntary memory as a function of memory retrieval phase across studies

Overall percentages of the memory recognition accuracy are presented in Fig. 1 and in Table 1. The overall means for the memory recognition accuracy were entered into a 2 groups (specified memory origin, unspecified memory origin) × 2 origin of memory (involuntary memory, voluntary memory) mixed ANOVA with repeated measures on the last factor. Neither the main effect of group, [*F*(1, 46) = 0.73, *p* > 0.397, $\eta_{{\text{p}}}^{2}$ = 0.02], origin of memory [*F*(1, 46) = 0.69, *p* > 0.411, $\eta_{{\text{p}}}^{2}$ = 0.01], nor the group by origin of memory interaction were significant [*F*(1, 46) = 1.46, *p* > 0.196, $\eta_{{\text{p}}}^{2}$ = 0.04]. Participants, only on rare occasions (i.e. only in 2% of cases for voluntary memories in both groups and 2% and 1% for involuntary memories in the specified memory and unspecified memory condition, respectively) were not able to answer the question and selected “*I do not know*” as an answer. This suggest that participants were comparable across conditions in remembering the origin of memories.

Finally, to control for the possible differences in the time-line of the experiment, the overall means for the length of the whole programme duration (the vigilance task, the voluntary memory recording phase, the break plus the post-task rating procedure) were compared between the two groups in an independent t-test. The groups (specified memory origin: *M* = 97.45 min., SD = 23.73; unspecified memory origin: *M* = 104.1 min., SD = 45.95) did not differ significantly from each other in this regard, *t(46)* = 0.63, *p* = 0.531, *d* = 0.20.

Therefore, we argue that any possible differences between groups in the phenomenological characteristics should not be due to group differences in the level the above-mentioned variables.

#### Strategy for analysing data on phenomenological characteristics

Entries designated by participants as autobiographical memories were included in the analysis. These memories were screened beforehand by two independent judges as memories or non-memories. The agreement between participants and judges for memories was perfect for both groups (i.e. 100%). For each participant, we calculated mean ratings, because subjects provided dependent multiple observations (for a detailed description see also Berntsen & Hall, 2004; Schlagman & Kvavilashvili, 2008). In addition, we calculated retrieval latencies (RT) for all IAMs reported by participants as triggered by a verbal phrase. As in previous studies (e.g. Barzykowski & Staugaard, 2016, 2018; Schlagman & Kvavilashvili, 2008), the RTs were calculated by adding up the time between the participant indicating a memory, and the onset of the verbal phrase which s/he had indicated as the trigger of that memory.^7^

We analysed differences between the two conditions in separate factorial ANOVAs with each memory characteristic as an outcome variable and intention (involuntary vs. voluntary) as within-subject factors.

Finally, while running these analyses we decided not to control for multiple comparisons since lowering the alpha value with any type of correction might actually help the hypothesis that layperson understanding of involuntary (IAMs) and voluntary memories (VAMs) does not influence the phenomenological characteristics of memories per se.

#### Frequency of memories

*The effects of intention (involuntary vs voluntary retrieval phase).* Participants recalled a total of 221 (*M* = 9.21, SD = 6.11, range 4–29) and 262 (*M* = 10.92, SD = 8.40, range 6–15) IAMs in the specified memory origin and the unspecified memory origin condition, respectively. At the same time, participants recalled 337 (*M* = 14.04, SD = 2.31, range 7–16) and 314 (*M* = 13.08, SD = 3.41, range 4–16) VAMs in the specified memory origin and unspecified memory origin condition, respectively.

As can be seen in Table 2, participants recalled significantly more memories in the voluntary phases compared to involuntary memory recording phases (main effect of intention, *F* (1, 46) = 9.89, $\eta^{2}$ = 0.18.

**Table 2:** The overall mean ratings for characteristics of memories as a function of intention and condition in Study 1

	Group 1: Specified Memory Origin				Group 2: Unspecified Memory Origin						
	Involuntary memories: Vigilance task (Report any spontaneously occurring memory)		Voluntary memories: Voluntary Autobiographical Memory task		Involuntary memories: Vigilance task (Report any spontaneously occurring memory)		Voluntary memories: Voluntary Autobiographical Memory task		Effects of intention (Voluntary vs Involuntary)	Effects of the memory origin (Specified vs. Unspecified origin of memory)	Interaction
	*M*	SD	*M*	SD	*M*	SD	*M*	SD	Test (Mixed ANOVA)		
Number of memories	9.21	6.11	14.04	2.31	10.92	8.40	13.08	3.41	***F (1, 46) = 9.89*** $\eta^{2}$ = 0.18*	*F* (1, 46) = .10 $\eta^{2}$** = **0.01	*F* (1, 46) = 1.44 $\eta^{2}$** = **0.03
Retrieval latencies	2.78	1.69	7.36	4.84	2.70	1.46	7.61	4.83	***F (1, 45) = 55.62*** $\eta^{2}$ = 0.55*	*F* (1, 45) = .01 $\eta^{2}$** = **0.01	*F* (1, 45) = .07 $\eta^{2}$** = **0.01
Online ratings											
Effort	2.23	0.76	3.02	1.19	2.42	1.12	3.00	1.17	***F (1, 46) = 18.57*** $\eta^{2}$ = 0.29*	*F* (1, 46) = .10 $\eta^{2}$** = **0.01	*F* (1, 46) = .42 $\eta^{2}$** = **0.01
Physiological sensation	3.56	1.32	2.77	1.19	3.20	1.10	2.48	0.92	***F (1, 46) = 29.90*** $\eta^{2}$ = 0.39*	*F* (1, 46) = 1.15 $\eta^{2}$** = **0.02	*F* (1, 46) = .07 $\eta^{2}$** = **0.01
Intensity of emotions	4.08	1.27	3.64	0.97	3.82	0.98	3.25	0.68	***F (1, 46) = 17.61*** $\eta^{2}$ = 0.28*	*F* (1, 46) = 1.56 $\eta^{2}$** = **0.03	*F* (1, 46) = .29 $\eta^{2}$** = **0.01
Vividness	4.69	0.88	4.26	0.75	4.62	0.69	4.05	0.74	***F (1, 46) = 23.96*** $\eta^{2}$ = 0.34*	*F* (1, 46) = .52 $\eta^{2}$** = **0.01	*F* (1, 46) = .50 $\eta^{2}$** = **0.01
Clarity	5.28	0.83	4.91	0.93	5.15	0.61	4.42	0.64	***F (1, 46) = 29.66*** $\eta^{2}$ = 0.39*	*F* (1, 46) = 2.53 $\eta^{2}$** = **0.05	*F* (1, 46) = 3.17 $\eta^{2}$** = **0.06
Specificity	4.81	0.93	4.51	0.91	4.71	0.72	4.10	0.94	***F (1, 46) = 15.32*** $\eta^{2}$ = 0.25*	*F* (1, 46) = 1.30 $\eta^{2}$** = **0.03	*F* (1, 46) = 1.77 $\eta^{2}$** = **0.04
Personal nature	4.16	0.88	3.66	1.11	4.18	0.94	3.32	1.06	***F (1, 46) = 18.01*** $\eta^{2}$ = 0.28*	*F* (1, 46) = .42 $\eta^{2}$** = **0.01	*F* (1, 46) = 1.29 $\eta^{2}$** = **0.03
Offline ratings											
Specificity ratio	0.53	0.17	0.57	0.19	0.62	0.23	0.57	0.21	*F* (1, 46) = .01 *η2* = .01	*F* (1, 46) = 76 $\eta^{2}$** = **0.02	*F* (1, 46) = 1.98 $\eta^{2}$** = 0**.04
Effort	1.97	0.66	2.91	1.03	2.62	0.91	3.00	0.91	***F (1, 46) = 33.40*** $\eta^{2}$ = 0.42*	*F* (1, 46) = 2.64 $\eta^{2}$** = **0.05	***F (1, 46) = 5.91*** $\eta^{2}$ = 0.11*
Specificity	4.57	0.80	4.35	0.86	4.79	0.47	4.15	0.62	***F (1, 46) = 29.19*** $\eta^{2}$ = 0.39*	*F* (1, 46) = .01 $\eta^{2}$** = **0.01	***F (1, 46) = 7.14*** $\eta^{2}$ = 0.13*
Vividness	4.64	0.77	4.28	0.75	4.86	0.63	4.30	0.66	***F (1, 46) = 28.43*** $\eta^{2}$ = 0.39*	*F* (1, 46) = .41 $\eta^{2}$** = **0.01	*F* (1, 46) = 1.50 $\eta^{2}$** = **0.03
Clarity	5.11	0.85	4.84	0.91	5.15	0.62	4.55	0.60	***F (1, 46) = 35.51*** $\eta^{2}$ = 0.44*	*F* (1, 46) = .35 $\eta^{2}$** = **.01	***F (1, 46) = 4.92*** $\eta^{2}$ = 0.10*
Personal nature	4.30	1.03	3.69	1.14	4.58	0.90	3.46	1.05	***F (1, 46) = 41.07*** $\eta^{2}$ = 0.47*	*F* (1, 46) = 01 $\eta^{2}$** = **0.01	***F (1, 46) = 3.68*** $\eta^{2}$ = 0.07*
Importance of the event	4.20	0.79	3.10	0.83	4.23	0.77	3.17	0.64	***F (1, 46) = 81.51*** $\eta^{2}$ = 0.64*	*F* (1, 46) = .07 $\eta^{2}$** = **0.01	*F* (1, 46) = .02 $\eta^{2}$** = **0.01
Valence of the memory	4.56	0.74	4.09	0.52	4.44	0.87	4.12	0.48	***F (1, 46) = 10.02*** $\eta^{2}$ = 0.18*	*F* (1, 46) = .10 $\eta^{2}$** = **0.01	*F* (1, 46) = .37 $\eta^{2}$** = **0.01
Identity	3.33	0.90	2.27	0.86	3.22	0.96	2.44	0.80	***F (1, 46) = 63.17*** $\eta^{2}$ = 0.58*	*F* (1, 46) = .02 $\eta^{2}$** = **0.01	*F* (1, 46) = 1.37 $\eta^{2}$** = **0.03
Intensity of emotions	3.87	0.89	3.06	0.85	3.93	0.90	3.12	0.80	***F (1, 46) = 66.57*** $\eta^{2}$ = 0.59*	*F* (1, 46) = .07 $\eta^{2}$** = **0.01	*F* (1, 46) = .01 $\eta^{2}$** = **0.01
Unusualness	4.00	0.63	3.05	0.80	4.10	0.57	3.17	0.52	***F (1, 46) = 85.24*** $\eta^{2}$ = 0.65*	*F* (1, 46) = .51 $\eta^{2}$** = **0.01	*F* (1, 46) = .01 $\eta^{2}$** = **0.01
Rehearsal: recalling in the past	3.39	0.69	2.65	0.71	3.55	0.68	2.70	0.58	***F (1, 46) = 58.38*** $\eta^{2}$ = 0.56*	*F* (1, 46) = .39 $\eta^{2}$** = **0.01	*F* (1, 46) = .30 $\eta^{2}$** = **0.01
The age of the memory	19.14	4.58	17.10	5.04	19.69	3.67	16.57	2.41	***F (1, 46) = 34.42*** $\eta^{2}$ = 0.43*	*F* (1, 46) = .01 $\eta^{2}$** = **0.01	*F* (1, 46) = 1.50 $\eta^{2}$** = **0.03

Please note that compared to Study 2, participants in the involuntary memory phase were instructed to report any spontaneously occurring memory

The online and offline phenomenological characteristics were rated on 7-point scales (1 = *low* to 7 = *high*). In the case of the age of the memory, participants indicated how old they were when the event in memory took place. Specificity ratio of memories was the proportion of specific relative to general memories

*p < 0.05; **p = 0.062 (i.e. Personal nature-interaction)

*The effects of information about the memory origin (specified memory origin vs unspecified memory origin group)* Neither the main effect of the memory origin nor the group by the memory of origin effect were significant. Since participants reported memories before the memory origin manipulation, we did not expect to see any effects of this manipulation on the number of memories.

#### Retrieval latencies of memories

*The effects of intention.* Table 2 shows that IAMs (*M* = 2.74, SD = 1.57, range 1.12–8.47) were retrieved significantly faster than VAMs (*M* = 7.43, SD = 4.74, range 1.54–21.30; *F* (1, 45) = 55.62, $\eta^{2}$ = 0.55).

*The effects of information about the memory origin.* We did not observe any significant main effects on retrieval latencies of the memory of origin nor the intention by origin of memory interaction.

#### Phenomenological characteristics of memories 

##### Characteristics recorded online

*The effect of intention* during the online-rating procedure, participants rated the physiological sensation, effort, intensity of emotions, vividness, clarity, specificity, and personal nature with each piece of IAMs and VAMs.

As Table 2 shows, we found a significant main effect of intention on all online ratings. More precisely, IAMs compared to VAMs were rated higher on all but one (i.e. effort—IAMs were rates as less effortfully retrieved) phenomenological characteristics (all *F’*s > 15.32).

*The effects of information about the memory origin.* Neither the main effect of the memory origin nor the intention by memory origin interaction were significant for the online ratings (all *F’*s < 3.17).

##### Characteristics recorded offline

*The effects of intention* During the post-task rating procedure, participants rated their recorded memories and recalled events on a number of additional phenomenological characteristics. As can be seen in Table 2, we observed a significant main effect of intention on all but one (i.e. specificity ratio) characteristic. More precisely, IAMs were rated higher on all but one (i.e. effort was lower for IAMs) characteristic compared with VAMs.

**Fig. 2:**
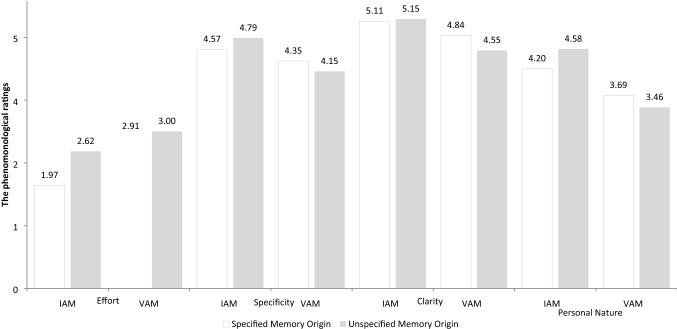
The effects of information about the memory origin on the phenomenological characteristics of memories in Study 1

*The effects of information about the memory origin* As shown in Table 2, while we did not observe a significant main effect of memory origin factor on any of the offline characteristics. The intention by memory origin interaction was significant for effort, specificity, clarity and personal nature (although this latter was only close to the statistical significance, *p* = 0.062). We present these results in Fig. 2. The post hoc tests showed that specifying the origin of a memory decreased the reported effort but only for IAMs. In addition, when the origin of the memory was specified the difference between IAMs and VAMs was: (1) smaller for specificity (*p* = 0.060 compared to *p* = 0.001), clarity (*p* = 0.011 compared to *p* = 0.001), personal nature (*p* = 0.003 compared to *p* = 0.001), and (2) bigger for effort (*p* = 0.001 compared to *p* = 0.022).

### Discussion

To investigate the role of metacognitive beliefs on the attribution of phenomenological characteristics to autobiographical memories, we manipulated the information given to participants when they were asked to rate the memories they had retrieved in an involuntary task and in a voluntary task. For half of the participants, the memories were presented in two separate blocks, clearly labelled as involuntary or voluntary. For the other half memories were presented in a mixed list without any labelling. Labelling the memories as voluntary and involuntary would activate and facilitate any existing metacognitive beliefs about involuntary and voluntary memories. If reporting phenomenological characteristics depends on metacognitive beliefs, then a main effect of information about the memory origin should be obtained on the characteristics assigned to the memories. For example, in the specified origin condition one might expect involuntary memories to be rated as more effortlessly retrieved, more clear, vivid, personal, important, to name just a few of possible expected differences.

In our overall results, we were able, first, to replicate the following well-known findings from previous studies (e.g. Barzykowski & Staugaard, 2016, 2018; Barzykowski et al., 2019a, b, c; Berntsen, Staugaard, & Sørensen, 2013; Schlagman & Kvavilashvili, 2008): (1) despite the fact that there were many more cues in the involuntary conditions, more voluntary memories were observed, (2) voluntary memories were retrieved significantly more slowly than involuntary memories, (3) involuntary memories compared to voluntary memories showed a range of indicators suggesting increased accessibility, including higher physiological impact, emotional intensity, vividness, clarity, specificity, personal nature, importance, positivity, unusualness, rehearsal and recency.

However, this pattern of results did not vary between the two groups, those who received information about the origin of the memory (voluntary vs involuntary task) and those who did not. This suggests that such subjectively attributed characteristics may reflect truly perceived properties of memories, without being influenced by any pre-existing metacognitive beliefs. While these findings are in line with the results of previous studies, they also support the hypothesis that involuntary retrieval (i.e. no intention and no expectancy that autobiographical memory will come to mind) favours highly accessible memories (threshold hypothesis, see Barzykowski & Staugaard, 2016, 2018; Barzykowski et al., 2019a, b, c). Therefore, as argued by Barzykowski et al. (2019a; b, c), Barzykowski and Staugaard (2016, 2018) involuntary memories may be an example of a memory content that, because of its phenomenological properties (e.g. emotional intensity, personal relevance, vividness, clarity, unusualness), are especially good at drawing one’s memory-related attention. As a result, they may pass the awareness threshold more easily and thus may more likely be reported. Attributions of phenomenological characteristics might then reflect the way people perceive their qualities during retrieval, rather than depending on preconceptions on how involuntary and voluntary memories should differ.

However, while we did not observe any main effects of the memory origin on phenomenological characteristics, we did find an interaction between the memory task (i.e. involuntary vs voluntary) and memory origin (i.e. specified vs unspecified origin). More precisely, involuntary memories recorded in the specified memory condition were rated with the lowest effort ratings compared to involuntary and voluntary memories across all conditions. This suggest that participants may have a preconception about how effortless involuntary memories should be, because they may believe that involuntary memories should pop up spontaneously and automatically.

It should be noted that, contrary to the hypothesis of belief influence, providing participants with information about the origin of the memory attenuated (but not eliminated) differences between involuntary and voluntary memories in terms of specificity, clarity and personal relevance. As a result, when participants did not know the exact origin of a memory, IAMs were rated as more specific compared to voluntary memories, more clear (clarity of voluntary memories was decreased) and, although shy of statistical significance, more of personal nature. These results can support the claim that the phenomenological characteristics of the two types of memories overall do not depend on a metacognitive belief on how voluntary and involuntary memories should be.

When discussing the results, it could be argued that instructing participants in the involuntary memory recoding phase to report only involuntary memories (i.e. engaging participants into monitoring their stream of awareness looking only for autobiographical memories) might have increased the similarity between involuntary and voluntary memories (for a similar argument see Batool & Mazzoni, 2011; Barzykowski & Niedźwieńska, 2016; Barzykowski & Staugaard, 2016, 2018; Barzykowski et al., 2019a, b, c; Vannucci et al, 2014). This could have influenced the phenomenological differences between involuntary and voluntary memories as well as other measures such as mental effort and reaction time. For this reason, we repeated the experiment while instructing participants, similarly to previous studies (e.g. Barzykowski & Staugaard, 2016, 2018, unrestricted conditions; Barzykowski & Staugaard, 2016, 2018; Barzykowski et al., 2019a, b, c, unrestricted conditions; Barzykowski & Niedźwieńska, 2018a, b; Mace & Unlu, 2020; Vannucci et al., 2014, unrestricted conditions), to report any spontaneously occurring thought during the involuntary memory recording phase.

## Study 2

The aim of Study 2 was to replicate the results of Study 1 using a so-called “unrestricted procedure” in the involuntary retrieval phase (e.g. Batool & Mazzoni, 2011; Vannucci et al 2014; Barzykowski & Niedźwieńska, 2016; Barzykowski & Staugaard, 2018). More precisely, during the unrestricted procedure, participants were instructed to report any mental content that popped into their minds and only later identify the memories among them. This minimizes the possibility that task demands and preliminary monitoring and selection affected the results (for discussions on the effect of retrieval monitoring, see Barzykowski & Niedźwieńska, 2016; Barzykowski & Staugaard, 2018; Barzykowski et al., , 2019a; b, c; Vannucci et al., 2014). We expected to replicate the main results of Study 1; namely: (1) there are more voluntary memories despite having more cues in the involuntary conditions, (2) voluntary memories are retrieved significantly more slowly than involuntary memories, (3) involuntary memories compared to voluntary memories demonstrate a range of indicators suggesting increased accessibility, (4) there were no robust and meaningful effects of metacognitive beliefs on the phenomenological characteristics of involuntary and voluntary memories. More specifically, if metacognitive beliefs indeed do not influence autobiographical memory retrieval, then the differences in phenomenological characteristics between involuntary and voluntary memories should be relatively stable across experimental conditions. Alternatively, if intuitive ideas of the qualities of involuntary and voluntary memories affect the memory retrieval, then we should observe stronger differences in phenomenological characteristics between involuntary and voluntary memories in the ‘specified memory origin’ group compared to the ‘unspecified memory origin’ group.

### Participants and method

A total of 60 participants (46 females, *M*_age_ = 23.78, SD = 3.96, range 19–36 years; two participants did not indicate their age) were recruited and randomly assigned to the two experimental groups: the specified memory origin and the unspecified memory origin. Participants were tested in groups of two to twelve in a laboratory with separate computer stations. Eight participants did not report any involuntary autobiographical memory, two had less than 50% of correct responses on the vertical lines task, and additional three participants guessed the true goal of the study (i.e. that we were specifically interested in memories), and their results were excluded from the analysis. Therefore, the final sample consisted of 47 participants with 22 participants in the specified memory origin condition (18 females, *M*_age_ = 23.80, SD = 4.02, range 19–35 years), and 25 participants in the unspecified memory origin condition (19 females, *M*_age_ = 23.20, SD = 2.74, range 20–30 years). They participated in return for a 20 PLN (ca. 5 USD).

The procedure was the same as in the Study 1. The only differences between the conditions in Study 1 and Study 2 were as follows: (1) participants were instructed to report any mental content that spontaneously entered their minds during the vigilance task (but they did not have to report task-related thoughts, e.g. *this is so boring, don’t forget to push the button, when it will finally end*), (2) after the completion of the vigilance task, participants answered open-ended questions concerning what they thought the true goal of the study was, (3) during the post-task rating procedure participants were asked to review all thoughts recorded during the vigilance task and decide which descriptions of mental content were autobiographical memories.

### Results

#### Equivalence of study groups

We investigated the comparability of research groups the same way as in Study 1. The results presented in Table 1 show that the groups did not differ from each other in terms of means for the SDS, the SSMQ, and the vigilance task performance (*p* > 0.246).

Similar to previous Study 1, the analysis on the mood scores measured by PANAS revealed a significant main effect of time of testing on the positive, *F*(1, 44) = 5.05, *p* < 0.030, $\eta_{{\text{p}}}^{2}$ = 0.10, and negative affect, *F*(1, 44) = 9.38, *p* < 0.004, $\eta_{{\text{p}}}^{2}$ = 0.18 showing that participants before the post-task rating procedure had lower ratings on both positive and negative affect scales compared to the beginning of the experimental session (see Table 1). Neither the main effect of group (origin of memory), nor the group by task (voluntary vs involuntary) interaction were significant (*p* > 0.064; *F* < 3.60).

In addition, neither the main effect of task, [*F*(1, 45) = 2.64, *p* > 0.111, $\eta_{{\text{p}}}^{2}$ = 0.06], origin of memory [*F*(1, 45) = 1.23, *p* > 0.274, $\eta_{{\text{p}}}^{2}$ = 0.03], nor the task by origin of memory interaction were significant [*F*(1, 45) = 0.01, *p* > 0.957, $\eta_{{\text{p}}}^{2}$ = 0.01]. Participants, only on rare occasions (i.e. only in 4% and 0% of cases for VAMs in the specified memory and unspecified memory origin, respectively, and in 1% for IAMs in both groups) were not able to accurately recognize the memory and selected “*I do not know*” as an answer. Therefore, we argue that participants were comparable across conditions in remembering the origin of memories.

Finally, groups (specified memory origin: *M* = 96.87 min., SD = 22.08; unspecified memory origin: *M* = 94.58 min., SD = 20.50) took an equal amount of time to complete the experiment *t*(*45*) = 0.21, *p* = 0.837, *d* = 0.11.

Therefore, we argue that groups were comparable and any differences in the phenomenological differences cannot be explained by the above-mentioned variables.

#### Strategy for data analysis

Entries designated by participants as autobiographical memories were included in the analysis. These memories were screened beforehand by two independent judges as memories or non-memories. The agreement between participants and judges for memories was perfect for the two groups (i.e. 100%); however, some of the thoughts indicated as autobiographical memories by the judges were not identified as memories by participants. As has been highlighted elsewhere (Barzykowski & Niedźwieńska, 2018b), since the decision whether a mental content was or was not a memory was irreversible, this might have resulted in some errors that the participants committed in the categorization task. In addition, after getting familiar with the post-task rating procedure, participants knew that the more memories they had, the longer the experiment would last. As the categorization task was performed at the very end of the experiment, this may have also affected their decisions in the categorization task. Therefore, similar to previous studies (e.g. Barzykowski et al., 2019a, b, c) re-evaluated entries (e.g. *the first time I kissed a girl*, *a memory of my father painting my room with me*) only with 100% agreement between judges were included in the analysis.

#### Frequency of memories

*The effects of intention* Participants recalled 144 (*M* = 6.55, SD = 6.56, range 1–26) and 159 (*M* = 6.36, SD = 6.85, range 1–31) IAMs in the specified memory origin and the unspecified memory origin groups, respectively. At the same time, participants recalled 264 (*M* = 12.00, SD = 3.68, range 2–16) and 332 (*M* = 13.28, SD = 3.06, range 3–16) VAMs in the specified memory origin and unspecified memory origin groups, respectively.

As can be seen in Table 3, participants recalled significantly more memories in the voluntary phases compared to involuntary memory recording phases (main effect of task, *F* (1, 45) = 45.42, *η*^2^ = 0.50).

**Table 3:** The overall mean ratings for characteristics of memories as a function of group in Study 2

	Group 1: Specified Memory Origin				Group 2: Unspecified Memory Origin						
	Involuntary memories: Vigilance task (Report any spontaneously occurring thought)		Voluntary memories: Voluntary Autobiographical Memory task		Involuntary memories: Vigilance task (Report any spontaneously occurring thought)		Voluntary memories: Voluntary Autobiographical Memory task		Voluntary vs Involuntary	Specified vs. Unspecified origin of memory	Interaction
	*M*	SD	*M*	SD	*M*	SD	*M*	SD	Test (Mixed ANOVA)		
Number of memories	6.55	6.56	12.00	3.68	6.36	6.85	13.30	3.06	***F (1, 45) = 45.42*** $\eta^{2}$ = 0.50*	*F* (1, 46) = .19 $\eta^{2}$ = 0.01	*F* (1, 46) = .64 $\eta^{2}$ = 0.01
Retrieval latencies	2.79	1.36	6.01	2.65	2.85	2.13	7.53	3.97	***F (1, 42) = 48.18*** $\eta^{2}$ = 0.53*	*F* (1, 46) = 1.69 $\eta^{2}$ = 0.04	*F* (1, 46) = 1.63 $\eta^{2}$ = 0.04
Online ratings											
Effort	2.18	1.00	3.31	1.40	1.98	1.14	3.23	1.48	***F (1, 45) = 32.47*** $\eta^{2}$ = 0.42*	*F* (1, 45) = .21 $\eta^{2}$ = 0.01	*F* (1, 45) = .07 $\eta^{2}$ = 0.01
Physiological sensation	3.63	1.44	2.62	1.10	3.14	1.27	2.68	1.18	***F (1, 45) = 16.20*** $\eta^{2}$ = 0.26*	*F* (1, 45) = .47 $\eta^{2}$ = 0.01	*F* (1, 45) = 2.28 $\eta^{2}$ = 0.05
Intensity of emotions	4.33	1.33	3.45	.84	4.21	1.05	3.63	0.96	***F (1, 45) = 17.43*** $\eta^{2}$ = 0.28*	*F* (1, 45) = .01 $\eta^{2}$ = 0.01	*F* (1, 45) = .73 $\eta^{2}$ = 0.02
Vividness	4.99	.77	3.95	1.22	4.64	1.08	4.13	0.89	***F (1, 45) = 22.49*** $\eta^{2}$ = 0.33*	*F* (1, 45) = .13 $\eta^{2}$ = 0.01	*F* (1, 45) = 2.65 $\eta^{2}$ = 0.06
Clarity	5.56	.83	4.57	.93	5.21	0.94	4.64	0.69	***F (1, 45) = 28.01*** $\eta^{2}$ = 0.38*	*F* (1, 45) = .46 $\eta^{2}$ = 0.01	*F* (1, 45) = 2.09 $\eta^{2}$ = 0.04
Specificity	4.91	1.21	4.14	0.93	4.69	1.02	4.35	0.90	***F (1, 45) = 11.73*** $\eta^{2}$ = 0.21*	*F* (1, 45) = .01 $\eta^{2}$ = 0.01	*F* (1, 45) = 1.88 $\eta^{2}$ = 0.04
Personal nature	4.50	1.70	3.65	0.99	3.93	1.50	3.44	1.18	***F (1, 45) = 10.73*** $\eta^{2}$ = 0.19*	*F* (1, 45) = 1.32 $\eta^{2}$ = 0.03	*F* (1, 45) = .78 $\eta^{2}$ = 0.02
Offline ratings											
Specificity ratio	0.36	0.29	0.59	0.24	0.46	0.33	0.61	0.15	***F (1, 45) = 14.82*** $\eta^{2}$ = 0.25*	*F* (1, 45) = 1.08 $\eta^{2}$** = **0.02	*F* (1, 45) = .58 $\eta^{2}$** = **0.01
Effort	2.39	1.67	3.44	1.78	2.26	1.39	3.19	1.38	***F (1, 45) = 23.34*** $\eta^{2}$ = 0.34*	*F* (1, 45) = .22 $\eta^{2}$** = **0.01	*F* (1, 45) = .09 $\eta^{2}$** = **0.01
Specificity	4.87	1.34	4.16	1.09	4.62	1.24	4.13	1.10	***F (1, 45) = 13.40*** $\eta^{2}$ = 0.23*	*F* (1, 45) = .21 $\eta^{2}$** = **0.01	*F* (1, 45) = .42 $\eta^{2}$** = **0.01
Vividness	5.30	1.07	4.40	1.00	4.70	1.14	4.16	0.97	***F (1, 45) = 27.08*** $\eta^{2}$ = 0.38*	*F* (1, 45) = 2.43 $\eta^{2}$** = **0.05	*F* (1, 45) = 1.71 $\eta^{2}$** = **0.04
Clarity	5.77	0.97	4.80	1.15	5.27	.98	4.65	0.79	***F (1, 45) = 32.76*** $\eta^{2}$ = 0.42*	*F* (1, 45) = 1.68 $\eta^{2}$** = **0.04	*F* (1, 45) = 1.57 $\eta^{2}$** = **0.03
Personal nature	4.70	1.66	3.89	1.43	3.91	1.70	3.61	1.33	***F (1, 45) = 7.88*** $\eta^{2}$ = 0.15*	*F* (1, 45) = 1.78 $\eta^{2}$** = **0.04	*F* (1, 45) = 1.64 $\eta^{2}$** = **0.04
Importance of the event	4.69	1.29	3.48	0.79	4.18	1.24	3.39	0.81	***F (1, 45) = 27.33*** $\eta^{2}$ = 0.38*	*F* (1, 45) = 1.54 $\eta^{2}$** = ** 0.03	*F* (1, 45) = 1.19 $\eta^{2}$** = **0.03
Valence of the memory	4.67	1.49	4.16	0.69	4.15	1.46	4.06	0.53	*F* (1, 45) = 1.88 $\eta^{2}$** = **0.04	*F* (1, 45) = 1.62 $\eta^{2}$** = **0.03	*F* (1, 45) = .91 $\eta^{2}$** = **0.02
Identity	3.75	1.55	2.56	1.01	2.89	1.40	2.11	0.77	***F (1, 45) = 22.08*** $\eta^{2}$ = 0.33*	***F (1, 45) = 5.25*** $\eta^{2}$ = 0.10*	*F* (1, 45) = 0.94 $\eta^{2}$** = **0.02
Intensity of emotions	4.75	1.22	3.46	.95	4.09	1.25	3.29	0.85	***F (1, 45) = 42.37*** $\eta^{2}$ = 0.48*	*F* (1, 45) = 2.37 $\eta^{2}$** = **0.05	*F* (1, 45) = 2.37 $\eta^{2}$** = **0.05
Unusualness	4.33	1.24	3.69	0.87	3.62	1.29	3.39	0.71	***F (1, 45) = 5.01*** $\eta^{2}$ = 0.10*	***F (1, 45) = 4.53*** $\eta^{2}$ = 0.09*	*F* (1, 45) = 1.11 $\eta^{2}$** = **0.02
Rehearsal: recalling in the past	4.11	1.40	2.93	0.77	3.47	1.29	2.90	0.61	***F (1, 45) = 18.87*** $\eta^{2}$ = 0.30*	*F* (1, 45) = 1.97 $\eta^{2}$** = **0.04	*F* (1, 45) = 2.30 $\eta^{2}$** = **0.05
The age of the memory	19.95	3.81	16.19	3.86	20.14	3.28	16.92	2.18	***F (1, 45) = 49.25*** $\eta^{2}$ = 0.55*	*F* (1, 45) = .26 $\eta^{2}$** = **0.01	*F* (1$\eta^{2}$** = ** = 0.01

Please note that compared to Study 1, participants in the involuntary memory phase were instructed to report any spontaneously occurring thought

The online and offline phenomenological characteristics were rated on 7-point scales (1 = low to 7 = high). In the case of the age of the memory, participants indicated how old they were when the event in memory took place. Specificity ratio of memories was the proportion of specific relative to general memories

^*^p < 0.05

*The effects of information about the memory origin* Neither the main effect of the memory origin nor the task by the memory of origin effect were significant additionally supporting the comparability of experimental groups.

#### Retrieval latencies of memories

*The effects of intention (task)* Table 3 shows that IAMs both in the specified memory origin and unspecified memory origin groups (*M* = 2.82, SD = 1.80, range 0.68–9.23) were retrieved significantly faster than VAMs (*M* = 6.70, SD = 3.46, range 1.58–16.77; *F* (1, 42) = 48.18, *η*^2^ = 0.53).

*The effects of information about the memory origin.* We did not observe any significant main effects of the origin of memory nor the task by memory of origin interaction.

#### Phenomenological characteristics of memories

##### Characteristics recorded online

*The effects of intention (task)* As Table 3 shows, we found a significant main effect of task on all online ratings. More precisely, IAMs compared to VAMs were rated higher on all but one (i.e. effort) phenomenological characteristics (all *F’*s > 10.73).

*The effects of information about the memory origin.* Neither the main effect of the memory origin nor the task by memory origin interaction were significant for the online ratings (all *F*’s < 2.63).

##### Characteristics recorded offline

*The effects of intention (task)* As can be seen in Table 3, we observed a significant main effect of intention on all but one (i.e. valence of the memory) characteristic compared with VAMs. More precisely, IAMs were rated higher on all but two (i.e. effort, specificity ratio were lower for IAMs) characteristic compared with VAMs.

*The effects of information about the memory origin* As shown in Table 3, while we did not observe a significant main effect of the task by memory origin interaction on any of the offline characteristics, the main effect of the memory origin for identity and unusualness were statistically significant. More precisely, memories (both IAMs and VAMs) recalled in the specified memory origin group were rated as more unusual and identity oriented compared to memories (IAMs and VAMs) reported in unspecified memory conditions.

### Discussion

While instructing participants to report any involuntarily retrieved mental content (i.e. not asking participants to monitor their flux of awareness looking only for memories), we replicated the findings of Study 1 demonstrating an increased accessibility of involuntary memories compared to voluntary memories (lower retrieval latencies and higher ratings of most of the phenomenological characteristics). Most pertinent, also in experiment 2 we did not observe any robust effects of being informed about the origin of the memory, although when participants knew the exact origin of memory they were more prone to rate all memories as more unusual and identity oriented compared to the unspecified memory group. Importantly, this effect was similar for involuntary and voluntary memories, suggesting that they indeed differ from each other in the perceived characteristics, rather than in memory-related beliefs.

## General discussion

In two studies we investigated the effects of metacognitive beliefs on the phenomenological properties of involuntary and voluntary autobiographical memories. Participants were randomized into two groups: specified memory origin and unspecified memory origin. A larger difference in characteristics between IAMs and VAMs in the group in which participants were told which memories had been retrieved in the involuntary task and which in the voluntary task would indicate that the main differences between involuntary and voluntary autobiographical memories reported in the literature are mainly due to the people’s naïve understanding of what involuntary and voluntary memories should be, rather how the memories are truly experienced.

In a broader sense, this issue relates also to a theoretical question about the extent to which the distinction between involuntary and voluntary retrieval is an attributional-based decision. In the present studies, we did not find a strong or convincing support to the notion that phenomenological memory characteristics are influenced by the knowledge of the origin of a memory. While we observed some main effects of memory origin, such knowledge did not change the observed differences between involuntary and voluntary memories reported in the literature (or it influenced equally both involuntary and voluntary memories). We discuss the main findings in more detail first, and then we further elaborate on their theoretical implications.

### Effects of intention (trying to retrieve a memory: involuntary vs voluntary memories)

Across two studies we were able to replicate the main findings reported in the literature (e.g. Barzykowski & Staugaard, 2016, 2018; Barzykowski et al., 2019a, b, c; Berntsen, Staugaard, & Sørensen, 2013; Schlagman & Kvavilashvili, 2008) demonstrating robust differences between involuntary and voluntary memories. More precisely, the presence of retrieval intentionality was mainly and directly manifested by the higher number of voluntary memories that took also longer to be retrieved compared to involuntary memories. In addition, consistently across both studies, involuntary memories were rated as retrieved with less effort and showed several indicators of increased accessibility.

These findings provide additional empirical support to the hypothesis that because of certain phenomenological properties (e.g. vividness, emotional intensity, clarity, personal relevance, unusualness), some mental contents may be especially good at drawing one’s memory-related attention and, thus, they may pass the awareness threshold more easily and are more likely to be reported (i.e. threshold hypothesis, Barzykowski & Staugaard, 2016, 2018; Barzykowski et al., 2019a, b, c; Barzykowski et al., 2020).

### The effects of information about the memory origin (whether retrieved in the involuntary or voluntary task)

When looking at the possible effects of the information about the memory origin, we expected to observe stronger differences between involuntary and voluntary memories in the ‘specified memory origin’ group, compared to the ‘unspecified memory origin’ group. We argued that if only metacognitive beliefs play a role, differences should be then minimized in the unspecified memory origin group and maximized in the specified memory origin group. In Study 1 where participants in the involuntary memory phase were instructed to report only spontaneously occurring memory, memories reported in the specified memory origin did not differ from memories in unspecified memory origin (i.e. main effects of the memory origin on phenomenological characteristics were not statistically significant). However, there was a significant interaction showing that knowing the origin of a memory made people rate involuntary memories as more effortlessly retrieved (compared to not knowing the origin of the memory). This result might be due to the information given in the specified memory origin condition, emphasizing that involuntary memories are retrieved automatically. If due to mentioning automaticity in retrieval, the effect also shows that the rating of memory characteristics can be relatively easily influenced, possibly by activating beliefs about characteristics of involuntary memories.

Finally, contrary to the hypothesis of belief influence, providing information about the origin of the memory actually attenuated (although not eliminated) differences between involuntary and voluntary memories in terms of specificity, clarity and personal relevance. Overall, then, these results cannot support the claim that the phenomenological characteristics of the two types of memories depend on the metacognitive belief on how voluntary and involuntary memories should be. Although there was an influence on perceived effort, the well-known robust differences between involuntary and voluntary memories observed in previous studies (e.g. Barzykowski & Staugaard, 2016, 2018; Barzykowski et al., 2019a, b, c; Schlagman & Kvavilashvili, 2008) were not abolished and/or undermined by the knowledge about the memory origin.

The same pattern of results was obtained in Study 2, in which participants in the involuntary memory phase were instructed to report any spontaneously occurring mental content. More precisely, while we did not observe any significant interaction, knowing the exact origin of a memory made participants more prone to rate memories in general as more unusual and identity oriented compared to the unspecified memory condition. This effect was similar for involuntary and voluntary memories, indicating that beliefs affected similarly both types of memories. Finally, it is important to highlight that we observed actually few effects of the memory origin across two studies, which supports the idea that the manipulating the presence/absence of information about the retrieval intentionality was generally effective.

Taking all these findings together, it can be argued that the differences between phenomenological characteristics of involuntary and voluntary memories seems then to reflect a genuine difference in qualities, that people perceive during retrieval, and are not due to memory-related beliefs.

### Theoretical implications

While the main goal of the present study was to examine the role of metacognitive beliefs on the phenomenological memory properties, the overarching goal was also to reflect on possible factors influencing involuntary and voluntary memory retrieval. Over the years, substantial knowledge has been gained about the way memories relating to our personal past are retrieved. The distinction between involuntary and voluntary memory retrieval was a main breakthrough (due to seminal work by Ebbinghaus, 1885 and pioneering studies by Berntsen, 1996, 1998), which led to substantial investigations (e.g. Barzykowski & Niedźwieńska, 2016, 2018a, b; Barzykowski & Staugaard, 2016, 2018; Barzykowski et al., 2019a, b, c; Berntsen, 1998; Berntsen et al., 2013; Mace, 2005; Mace & Unlu, 2020; Mazzoni, 2019; Mazzoni et al., 2014; Plimpton, Patel, & Kvavilashvili, 2015; Schlagman & Kvavilashvili, 2008; Staugaard, & Berntsen, 2014; Vannucci et al., 2014, 2015). At the same time, different types of voluntary memories (directly and generatively retrieved) were identified (Conway & Pleydell-Pearce, 2000; Harris et al., 2015; Haque & Conway, 2001; Rubin & Berntsen, 2009; Uzer et al., 2012) which differ in terms of how automatically and effortlessly they are retrieved. We underline the fact that this distinction already represents an attenuation of the strong division between involuntary and voluntary memories. Recently, Barzykowski and colleagues (Barzykowski & Staugaard, 2016, 2018; Barzykowski et al., 2019a, b, c) went even further and addressed the possibility that the retrieval of voluntary direct memories and involuntary memories might be relatively similar. In other words, involuntary retrieval shares several processes and stages with voluntary direct retrieval, with differences pertaining mostly to the intentionality of the initial search. The authors proposed the *threshold hypothesis* to explain the complex interplay between different factors operating at several levels of memory retrieval. For additional clarity, let us unfold the act of a given autobiographical memory retrieval into the following stages (Wilckens, Erickson, & Wheeler, 2012)^8^: (1) pre-retrieval stage, (2) retrieval stage, (3) post-retrieval stage, (4) retrieval outcome report stage. (1) Various processes, including the creation of expectations, during the pre-retrieval stage can boost or impair the retrieval stage. For example, one may be placed in a “*retrieval mode*” in which “*the cognitive system is prepared for or expects memory construction and recollection*” (Conway, 2001, p. 1379). During this phase retrieval intentionality can also be explicitly formed influencing the subsequent retrieval process. The effect of priming might also occur in this phase, as it changes the cue-item discriminability, which can be defined as “how easily a given cue isolates an item” (Rubin, 1995, p. 151 as cited in Berntsen, 2009, p. 107), and, for some memories, it enhances the likelihood that the memory will enter a person’s awareness. (2) The retrieval stage relates to the forming and developing of an autobiographical memory, but without explicit self-reflection; namely, a given memory might have been formed but one may not be explicitly aware of having a memory yet (something that refers to an *experiential level of consciousness*; Baird et al. 2013). Importantly, during this stage a memory is triggered by and/or accessed via a given cue, and it may be either reconstructed, directly retrieved, voluntarily searched or involuntarily recalled, depending on the memory pre-retrieval and retrieval processes involved. (3) After the memory is formed, during the post-retrieval stage people may realize (i.e. by monitoring their stream of awareness, and/or mental contents) to have a memory in mind. Thus, this stage relates to the ability to, for example, extract autobiographical content from the stream of consciousness to explicitly become aware of having a memory that is autobiographical (this is the level of meta-awareness). One may also control the retrieval process by directing or modifying it, depending on the goal (e.g. if the goal of the retrieval is or is not achieved in the voluntary retrieval). At this stage, then one is fully aware that an autobiographical memory was actually retrieved, and in voluntary retrieval, if the memory content meets the given criteria then the search may be terminated. (4) In the last stage, the retrieved memory may be shared with others and reported by giving a verbal account of the content. This approach then focuses on two main difference between involuntary and voluntary memories, the first being intentionality during the pre-retrieval phase, the second being metacognitive monitoring and control processes and beliefs (see Koriat & Goldsmith, 1996; Mazzoni & Kirsch, 2002), which may play a role in all four phases.

Previous studies provide support to the notion that these stages may serve as independent, albeit interconnected, retrieval phases. For example, Baird and colleagues (Baird et al., 2013) demonstrated that the participant’s ability to monitor their stream of awareness and extract content of thoughts from it (including autobiographical contents) may be impaired by cognitively demanding tasks. As a result, it can occur that even when a memory is successfully retrieved (retrieval stage), one might not be aware of having it in mind (post-retrieval stage).^9^ As a result, involuntary memories are less frequently observed under highly demanding activities (e.g. Barzykowski & Niedźwieńska, 2018b; Vannucci et al, 2015, 2019).

It may be argued that meta-cognitive beliefs and preconceptions about different types of memories may operate during pre-retrieval, post-retrieval and retrieval outcome report stages. For instance, as argued by Vannucci et al. (2014), having a clear expectation of what an autobiographical memory should be, one may decide in the pre-retrieval phase to look only for this type of memories; similarly, in the post-retrieval phase memories are selected that meet more closely some given criteria. There is also the possibility that some pre-existing beliefs typical of the post-retrieval phase might retroactively influence the pre-retrieval phase, by priming a certain type of memories, thus increasing their possibility to be retrieved. Similarly, but with opposite effects, participants beliefs about what a memory should be can influence negatively its report. For example, the memory plausibility criteria influence what people report as memories (e.g. it is possible, by changing the belief of the plausibility of demonic possession can change their rate of report, Mazzoni et al, 2001; Mazzoni, 2007). One can also hypothesize that when reporting the properties of a given memory, one may boost or downplay its properties, depending on the idea about how these memories should be (retrieval outcome reporting phase).

While the memory retrieval as a whole should not be treated as an attribution-based process, some aspects should be. For example, processing its outcomes, reflecting on the memory properties, and eventually reporting them may indeed take such an attributional form. This accords well with the Whittlesea’s approach (e.g. Whittlesea, 1997; Whittlesea & Leboe, 2000) positing that subjective judgements and evaluations are rather independent and separate from retrieval (production) and can influence how this retrieval proceeds (Mazzoni & Hanczakowski, 2011). For this reason, one should take into account the role of attributions when studying such a transient form of remembering as involuntary memories. Although in the current study we found that overt, belief/based attributional processes have only a minimal role in the retrieval of involuntary memories, dividing the retrieval act into smaller sub-phases opens up a set of interesting questions, also for future research on autobiographical memory retrieval.

### Possible limitations and future directions

When discussing the results of the present studies, some limitations may be taken into account. For example, while engaging our participants into the involuntary and voluntary memory retrieval phase we did not counterbalance the order of these conditions. As a result, participants first performed the vigilance task (i.e. involuntary memory retrieval phase) and then they were instructed to recall autobiographical memories in response to each cue presented on the screen. This was mainly because we wanted to keep our participants unaware of the true goal of our study; namely, the autobiographical memory retrieval. Engaging participants into the voluntary memory retrieval before the involuntary memory phase might have maximized the risk that participants during the involuntary retrieval phase would continue voluntarily recalling memories in response to cues presented on the screen.

One may also argue that the delay (i.e. 40 min) between the online-rating procedure of the involuntary retrieval phase and post-rating procedure was insufficient to make participants rely on metacognitive beliefs in the condition in which they had been informed about the voluntary or involuntary origin of the memory. With a longer delay the memory for the origin of the memory would have faded more, leaving more room for an effect of metacognitive beliefs (participants would have to rely more on the intuitive ideas of the qualities that should characterize involuntary and voluntary memories). Thus, it would be interesting to extend the interval to a few days.

It may be also argued that engaging participants in the online-rating procedure might also have an anchoring effect on participants and thus limit how different people would then rate their memories during the post-task rating procedure. We however feel this to be rather unlikely, given the number of rating questions per each mental content and the delay period. In addition, this would be especially unlikely in Study 2, where during the involuntary rating procedure participants rated any involuntarily occurring mental contents and then, during the post-task rating procedure answered questions in relation only to autobiographical memories. Since there were more additional mental contents, remembering each memory origin was even more difficult in Study 2 than in Study 1.

It is also worth highlighting that in our studies we did not explicitly ask our participants about their metacognitive beliefs of involuntary and voluntary memories and retrieval. Thus, there is still a need for more direct measure of metacognitive beliefs (e.g. Carciofo et al., 2017; Zedelius et al., 2020). For example, it would be interesting to examine what pre-existing beliefs participants may actually have of different types of memories and then investigate how these beliefs do actually influence the memory retrieval. Finally, as already mentioned in the introduction, the rationale of the study was that if only metacognitive beliefs influence the rating of memory characteristics, then we should observe larger differences between involuntary and voluntary memories when participants are highly focused on this distinction. However, one may argue that it would be important to verify first which beliefs people hold about the characteristics of involuntary and voluntary memories. While there are no published studies on this matter yet, recently Sanson, Staugaard, & Barzykowski, in preparation asked two groups of participants to rate the phenomenological characteristics of typical involuntary or voluntary memory. Although participants were not instructed to recall their own memory but only to rate general characteristics, involuntary compared to voluntary memories were rated as less effortful, less voluntary, more interrupting the ongoing activity, more mood changing, less positive and more negative, more often accompanied by physical reactions, more unusual, and with more intense emotions. These findings clearly demonstrate that people may indeed have some sort of layperson understanding of what involuntary and voluntary memories should be. Therefore, we argue that such pre-existing metacognitive beliefs should show an effect on the rating of characteristics of memories people retrieve. Future studies will address the relationship between pre-existing beliefs and the rating of memory characteristics.

### Final conclusions

In the present studies we investigated the possible role of metacognitive beliefs on the phenomenological characteristics of involuntary and voluntary autobiographical memories. Across two studies, our results clearly and consistently demonstrated that the phenomenology of the memories in general, and the phenomenological differences between involuntary and voluntary memories, in particular, are not strongly and relevantly influenced by metacognitive beliefs. Involuntary memories were rated as very different from voluntary memories, replicating previous findings showing the increased accessibility of involuntary memories. Taking these results together, the two studies suggest that involuntary memories might indeed have intrinsic characteristics that make them phenomenologically different from voluntary memories, differences which are not due to people’s understanding of what involuntary and voluntary memories should be.

## Footnotes

1 The term *threshold* may be defined as “*the minimal amount of activation required to become consciously aware of a stimulus*” (Reed, 2007, p. 49). Thus, a stimulus below the threshold of conscious awareness will not affect and enter consciousness and will not be consciously experienced.

2 We decided to use the term phenomenologically “*juicy*” as a figure of speech to highlight and summarize general properties of highly accessible memories that for this reason can pass the awareness threshold easily and are therefore able to enter consciousness even without one’s intention (i.e. involuntarily).

3 Of course, accessibility of a memory should be considered in relative terms. While a given memory may be sufficiently activated to pass the awareness threshold when one tries to retrieve it (intention), the same memory may not be activated strongly enough to pass the awareness threshold involuntarily, especially if one is not in a retrieval mode.

4 Importantly, as Zedelius et al (2020) highlighted the differences between metacognitive beliefs about a given phenomenon in general (i.e. what do people think of differences between IAMs and VAMs in general) and an individual assessment of one’s own abilities (i.e. what do people think of their own IAMs and VAMs), in the present study when discussing the metacognitive beliefs we need to clarify that we address the former ones, namely, people’s understanding of the IAMs and VAMs phenomena in general.

5 But importantly, more studies on the way participants classify involuntary and voluntary memories and what are the laypeople’s theories about memory retrieval, in general, and involuntary and voluntary memory retrieval, in particular, are now under preparation (e.g. Barzykowski, Staugaard, & Mazzoni, 2021; Sanson, Staugaard, & Barzykowski, in preparation).

6 The question relating to participants’ classification of memories as either involuntary or voluntary was asked for another purpose and the results are reported and discussed elsewhere (see Barzykowski, Staugaard, & Mazzoni, 2021).

7 More precisely, it was calculated as Total RTs = RTs + (2 s × Nos), where RTs are the milliseconds between the presentation of the closest cue and the reporting of a memory, 2 s is the constant presentation time for each slide, and Nos is the number of slides between the trial when a participant indicated having a memory and the slide with the verbal phrase which the participant indicated triggered that memory. For example, knowing that the memory ‘attending my sister’s wedding’ was (a) written 1.2 s after the presentation of a slide, and that (b) the cue ‘wedding’ that triggered the memory was 3 slides before the cue when the memory was reported, the retrieval latency equals to 7.2 s (1.2 s + 3 × 2 s).

8 Please note that these stages are demonstrative and are presented to better disentangle the possible factors operating during a memory retrieval. We think of these stages as a dynamic system, where the retrieval process can jump back and forth and even activate several stages at any given time. A helpful analogy is a neural network.

9 Baird et al. (2013) used probe-caught method and self-caught method combined together and observed that the target thoughts were also caught using the probe-caught method although they were not reported in self-caught reports.
